# Record linkage in tuberculosis epidemiological surveillance in Brazil: systematic methodological literature review

**DOI:** 10.1590/1980-549720260045

**Published:** 2026-08-24

**Authors:** Klauss K. S. Garcia, Amanda Amaral Abrahão, Henry Maia Peixoto, Elisabeth Carmen Duarte, Wildo Navegantes de Araújo, Walter Massa Ramalho

**Affiliations:** I London School of Hygiene and Tropical Medicine, Faculty of Epidemiology and Population Health, Department of Infectious Disease Epidemiology and International Health – London, United Kingdom.; II Universidade de Brasília, Tropical Medicine Center – Brasília (DF), Brazil.; III Universidade de Brasília, School of Health Sciences – Brasília (DF), Brazil.; IV Universidade de Brasília, School of Sciences and Health Technology – Brasília (DF), Brazil.

**Keywords:** Record linkage, Health Information Systems, Tuberculosis, Public Health Surveillance, Systematic Review

## Abstract

**Objective:**

To describe the literature on the methodological characteristics and use of record linkage techniques applied to tuberculosis surveillance in Brazil.

**Methods:**

A systematic methodological review (PROSPERO: CRD42024623969) was conducted following the 2020 guidelines of the Preferred Reporting Items for Systematic Reviews and Meta-Analyses (PRISMA). Searches were performed on the following databases: PubMed, Embase, Scopus, Virtual Health Library (VHL), Scientific Electronic Library Online (Scielo), and Latin American and Caribbean Literature on Health Sciences (LILACS) on February 5, 2025. Scientific articles produced in Brazil and whose authors applied record linkage between health information systems for epidemiological surveillance purposes were included. Grey literature, studies without a focus on tuberculosis, studies without application of linkage, and studies conducted outside Brazil were excluded. Descriptive synthesis was used, reporting frequencies, proportions, and measures of central tendency. Risk of bias or methodological quality were not assessed due to the heterogeneity of study designs and objectives, and because this review aimed to describe the methodological characteristics of the studies rather than synthesize effect estimates or epidemiological associations.

**Results:**

The search retrieved 1,549 records; 49 studies were included. Most studies used the tuberculosis module of the Notifiable Diseases Information System (SINAN-TB, 98.0%) and the Mortality Information System (SIM, 67.3%); the most frequently used software was OpenRecLink (57.1%). The most common key variables were "name," "date of birth," and "mother's name." The linkage percentage ranged from 0.1 to 97.3%. The most frequent objectives were the description of epidemiological scenarios (24.5%), analysis of associated factors (20.4%), and investigation of underreporting (16.3%). Cohort (49.0%) and descriptive (34.7%) study designs predominated.

**Conclusion:**

Record linkage integrates complementary databases and may strengthen tuberculosis surveillance. The findings suggest the potential usefulness of record linkage to support epidemiological analyses, improve records, and inform surveillance actions.

## INTRODUCTION

Tuberculosis is one of the main infectious diseases in the world and in Brazil, with a high incidence especially in regions with severe socioeconomic inequalities^1^. Brazil is among the nations with the highest tuberculosis burden in the world^1^, with incidence rates above 37 cases every 100 thousand inhabitants and over 80 thousand new cases of tuberculosis per year since the new coronavirus (COVID-19) pandemic^2^.

To eliminate tuberculosis, Brazil has adopted a series of measures provided for in the EndTB Strategy, which aims to eliminate the disease in the country by 2035. This strategy is aligned with the Sustainable Development Goals (SDGs), which, in its Target 3.3, aims at ending the epidemics of infectious and parasitic diseases, including tuberculosis, by 2030^1,3,4^.

Among the various issues that influence the process of eliminating tuberculosis in Brazil, the challenges of using Brazilian Health Information Systems (HIS) can be highlighted, such as the Notifiable Diseases Information System (*Sistema de Informação de Agravos de Notificação* – SINAN) and the Mortality Information System (*Sistema de Informação sobre Mortalidade* – SIM), which do not have interoperability, making it difficult, for instance, to jointly monitor cases of tuberculosis and deaths^5–7^.

To overcome this technological limitation, record linkage techniques are used for relating the HIS. This process consists of identifying and combining records that correspond to the same individual in different databases, even when there is no common unique identifier, which could be the Taxpayer Registration Number (*Cadastro de Pessoa Física* – CPF) or the National Health Card (SUS [Brazilian Unified Health System] Card)^7^.

Within the context of tuberculosis epidemiological surveillance, linkage allows a more complete view of the clinical history of patients and the outcomes^8,9^. In addition, these techniques help detect and eliminate duplicate records (deduplication technique) by consolidating non-repeated notifications^10,11^. Overall, the process involves previous steps of data preparation, such as cleaning, standardization and, in some studies, blocking, with the aim of making linkage more feasible and efficient^12^.

Considering that the use of record linkage techniques is widespread, especially in the context of tuberculosis surveillance, in this systematic review, we aim to describe the methodological characteristics and the use of linkage techniques applied to studies on tuberculosis in Brazil—and, based on these findings, to discuss how these techniques are used and can be used for supporting the epidemiological surveillance of tuberculosis.

## METHODS

This systematic methodological review was registered on the International Prospective Register of Systematic Reviews (PROSPERO) platform under identifier CRD42024623969 and developed according to the 2020 guidelines of the Preferred Reporting Items for Systematic Reviews and Meta-Analyses (PRISMA).

### Context

The study is inserted in the Brazilian context according to the following research question: "How have record linkage techniques been used in epidemiological studies on tuberculosis in Brazil?", mainly focusing on the application of record linkage techniques from HIS used by epidemiological surveillance of tuberculosis in the country.

### Study design

This is a systematic methodological literature review seeking to describe methodological characteristics of studies that applied record linkage techniques for tuberculosis surveillance in Brazil. Moreover, we sought to evaluate the contexts for the application of these techniques, the challenges faced, and the opportunities resulting from their use in the HIS.

### Search strategies and databases

The final search was carried out on February 5, 2025 in six electronic databases: PubMed, Embase, Scopus, Virtual Health Library (VHL), Scientific Electronic Library Online (Scielo), and Latin American and Caribbean Literature on Health Sciences (LILACS). The strategies were adapted for each database and controlled descriptors and free terms related to record linkage, tuberculosis, and Brazil were combined with explicit use of Boolean operators. In Table 1, the terms used and their combination structure are presented according to database.

**Table 1:** Search strategies used.

Database	Strategy
PubMed	(("linkage" OR "linked" OR "Medical *Record* linkage "[Mesh] OR "Medical *Record* linkage " OR "Health *Record* linkage " OR "*Record* linkage " OR "Data linkage " OR "Centre for Data and Knowledge Integration for Health" OR "CIDACS") AND (Brazil OR Brazilian) AND (Tuberculosis))
Embase	‘tuberculosis’ AND [embase]/lim AND (‘brazil’ OR ‘brazilian’) AND [embase]/lim AND (linkage OR ‘linked’ OR ‘medical *Record* linkage /exp OR ‘medical *Record* linkage OR ‘health *Record* linkage OR ‘*Record* linkage OR ‘data linkage OR ‘centre for data and knowledge integration for health’ OR ‘cidacs’) AND [embase]/lim AND ‘article’/it
Scopus	ALL("linkage " OR "linked" OR "medical *Record* linkage " OR "health *Record* linkage " OR "*Record* linkage " OR "data linkage " OR "centre for data and knowledge integration for health" OR "CIDACS") AND TITLE-ABS-KEY(tuberculosis) AND AFFILCOUNTRY("Brazil") AND DOCTYPE("ar") AND NOT (SUBJAREA("IMMU") OR SUBJAREA("BIOC") OR SUBJAREA("AGRI"))
VHL	("vinculação" OR "vinculado" OR "vinculação de registros médicos" OR "vinculação de dados" OR "ligação de registros médicos" OR "CIDACS" OR "vinculación" OR "vinculado" OR "vinculación de registros médicos" OR "vinculación de datos" OR "enlace de registros médicos" OR " linkage " OR "linked" OR "medical *Record* linkage " OR "health *Record* linkage " OR "*Record* linkage " OR "data linkage " OR "Centre for Data and Knowledge Integration for Health") AND ("Brasil" OR "brasileiro" OR "brasileira" OR "brasileiros" OR "brasileiras" OR "Brasil" OR "brasileño" OR "brasileña" OR "brasileños" OR "brasileñas" OR "Brazil" OR "Brazilian") AND ("tuberculose" OR "tuberculosis")
SciELO	("vinculação" OR "vinculado" OR "vinculação de registros médicos" OR "vinculação de dados" OR "ligação de registros médicos" OR "CIDACS" OR "vinculación" OR "vinculado" OR "vinculación de registros médicos" OR "vinculación de datos" OR "enlace de registros médicos" OR " linkage " OR "linked" OR "medical *Record* linkage " OR "health *Record* linkage " OR "*Record* linkage " OR "data linkage " OR "Centre for Data and Knowledge Integration for Health") AND ("Brasil" OR "brasileiro" OR "brasileira" OR "brasileiros" OR "brasileiras" OR "Brasil" OR "brasileño" OR "brasileña" OR "brasileños" OR "brasileñas" OR "Brazil" OR "Brazilian") AND ("tuberculose" OR "tuberculosis" OR "tuberculose" OR "tuberculose")
LILACS	("vinculação" OR "vinculado" OR "vinculação de registros médicos" OR "vinculação de dados" OR "ligação de registros médicos" OR "CIDACS" OR "vinculación" OR "vinculado" OR "vinculación de registros médicos" OR "vinculación de datos" OR "enlace de registros médicos" OR " linkage " OR "linked" OR "medical *Record* linkage " OR "health *Record* linkage " OR "*Record* linkage " OR "data linkage " OR "Centre for Data and Knowledge Integration for Health") AND ("Brasil" OR "brasileiro" OR "brasileira" OR "brasileiros" OR "brasileiras" OR "Brasil" OR "brasileño" OR "brasileña" OR "brasileños" OR "brasileñas" OR "Brazil" OR "Brazilian") AND ("tuberculose" OR "tuberculosis")

### Study location and population

The study focuses on research carried out in the Brazilian context whose authors employed record linkage techniques applied to the epidemiological surveillance of tuberculosis, without restrictions concerning time, location, or population.

### Inclusion and exclusion criteria

Scientific articles addressing the use of record linkage techniques in epidemiological investigations on tuberculosis in Brazil were included. Studies that used data from Brazilian health information systems and implemented record linkage procedures were considered eligible.

In turn, studies whose main focus was not tuberculosis; carried out outside Brazil; without direct application of record linkage for epidemiological surveillance purposes; or studies in a format other than scientific articles—such as conference abstracts, technical reports, and institutional documents—were excluded.

### Selection process

The references of the six databases were exported to Rayyan, consolidated and deduplicated. Two researchers screened titles and abstracts according to predefined criteria. Potentially eligible records were read in full, with reapplication of the criteria and exclusion of remaining duplicates. The selection was independent between the two researchers.

In addition to the electronic search, a manual search was performed in the bibliography of the included studies, with the objective of identifying potentially eligible articles not retrieved in the initial strategies. The identified studies were evaluated according to the same eligibility criteria and, where relevant, incorporated into the final set.

### Data extraction and analysis

Data extraction was performed independently by two researchers to ensure consistency and quality. The extracted variables were: title, main objective, year and place of publication, language, study period, type of linkage (probabilistic or deterministic), use of blocking, key variables of record linkage, involved HIS, and software used. For data tabulation and analysis, the Microsoft Excel software was used, while Python language^13^ was used for producing graphs and visual representations of the results. There was no formal evaluation of methodological quality or risk of bias due to the heterogeneity of the study designs, analytical objectives, and forms of reporting of the linkage procedures in the included studies.

#### Data Availability Statement:

All data used in this review were extracted from published scientific articles and are described in the methods section and Supplementary Table 1. No individual-level, confidential, or unpublished data were used.

## RESULTS

**Figure 1:**
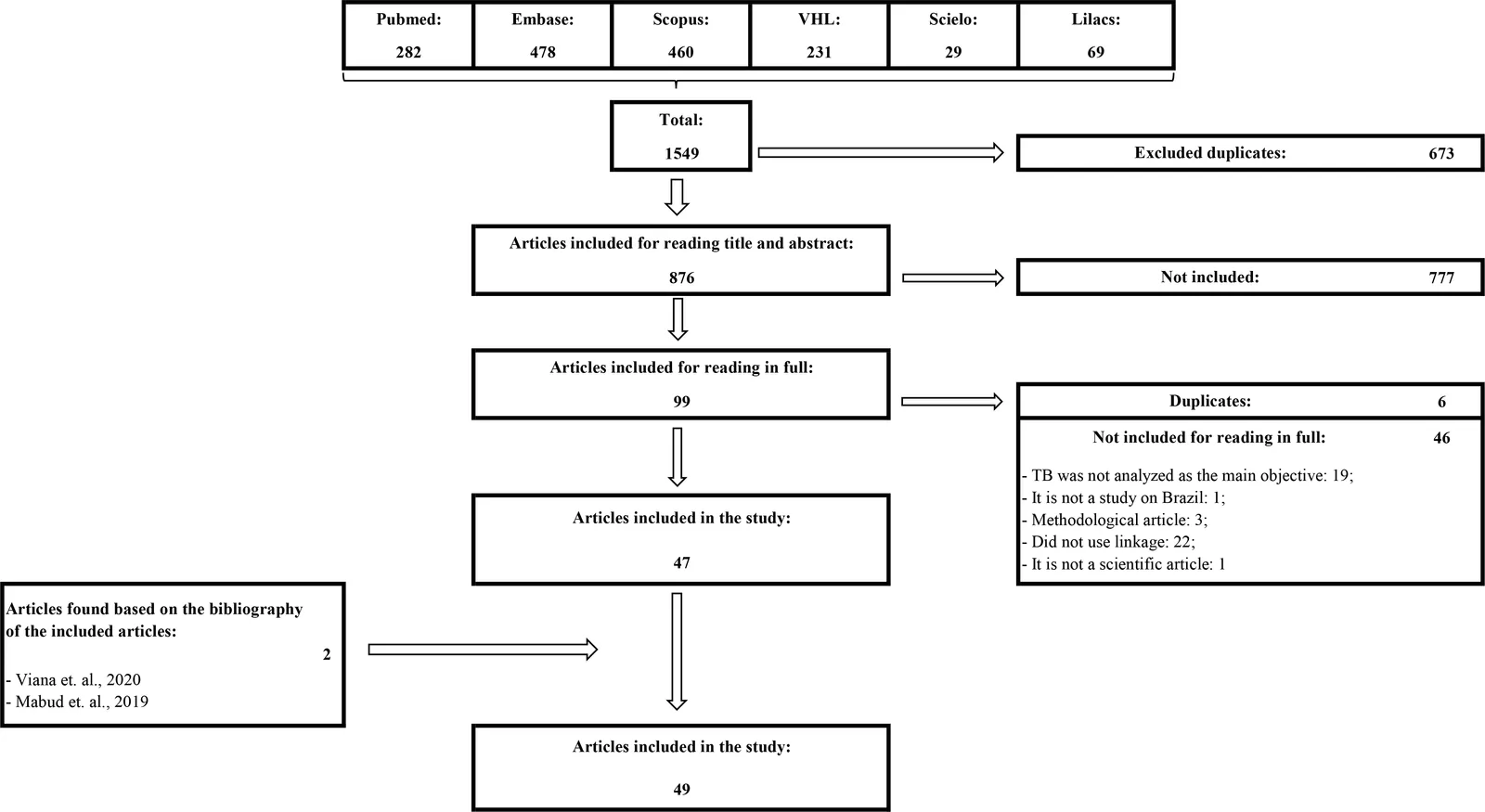
Flowchart of the selection process of the articles included in the review.

The initial search resulted in 1,549 articles, of which 678 were identified as duplicates and excluded. After reading titles and abstracts, 777 records were excluded, with 94 remaining for reading in full. After evaluating the texts in full, 47 articles were included. Subsequently, two additional studies identified by manual search in the bibliography were incorporated, totaling 49 included studies (Figure 1).

**Figure 2:**
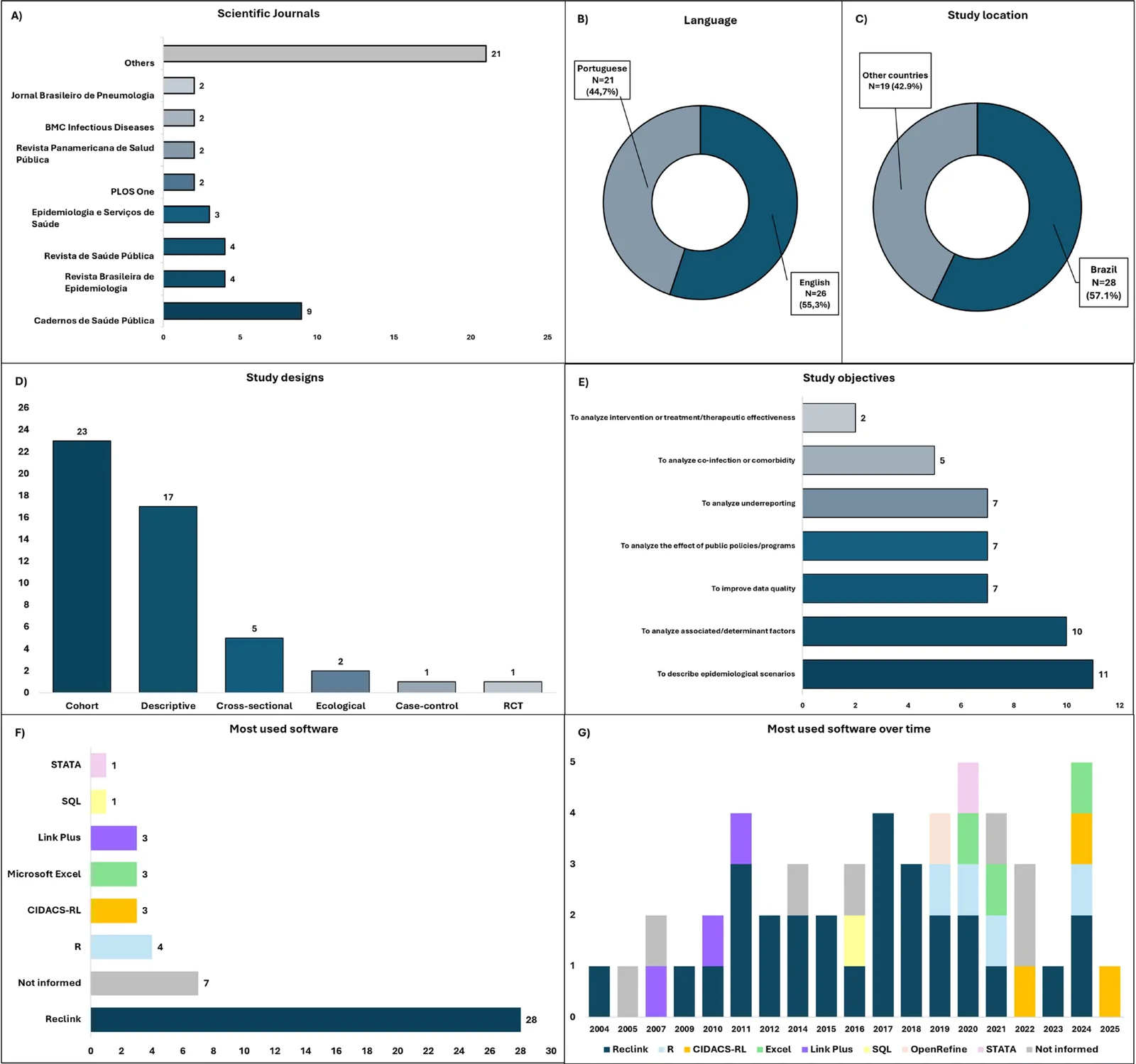
Main characteristics of the included articles.

The articles were published from 2004 to February 2025, the average of publications was 2.2 articles per year, and in the years 2006, 2008, and 2013 no eligible publications were identified. The journal with the highest number of publications on the subject was *Cadernos de Saúde Pública* [Reports in Public Health] (n=10; 20.4%), followed by *Revista Brasileira de Epidemiologia* [Brazilian Journal of Epidemiology] (n=4; 8.2%) and *Revista de Saúde Pública* [Journal of Public Health] (n=4; 8.2%) (Figure 2A). Most articles were published in national journals (n=29; 59.2%) (Figure 2B), but mostly in English (n=28; 57.1%) (Figure 2C).

Of the 49 articles, 24 were cohort studies (49%), 17 were descriptive studies (34.7%), and five were cross-sectional studies (10.2%) (Figure 2D). Of the 49 articles, the most frequent objective was to describe epidemiological scenarios (n=12; 24.5%), followed by analyzing factors associated with tuberculosis-related outcomes (n=10; 20.4%), and analyzing the underreporting of tuberculosis in HIS (n=8; 16.3%) (Figure 2E). Furthermore, linkage was also used to analyze scenarios of co-infections and impacts of public programs and policies on the incidence of tuberculosis (Figure 2E).

The most used software was OpenRecLink (version 3) (n=28; 57.1%), followed by R (n=4; 8.2%), and Cidacs-RL, Linkplus, and Microsoft Excel (three articles for each software; 6.1%), while in eight articles (16.3%) the used software was not reported (Figure 2F). We observed that the use of RecLink remained constant over the years and that, as of 2019, there was a greater diversity of software, including R and the Cidacs-RL algorithms (Figure 2G).

The key variables most frequently used were the combination of "patient's name" (n=39 articles; 79.6%), "date of birth" (n=39 articles; 79.6%), and "mother's name" (n=34 articles; 69.4%), while in ten articles (20.4%) the key linkage variables used were not informed. Other articles, less frequently, also used "sex" and "address" information as key variables.

**Figure 3:**
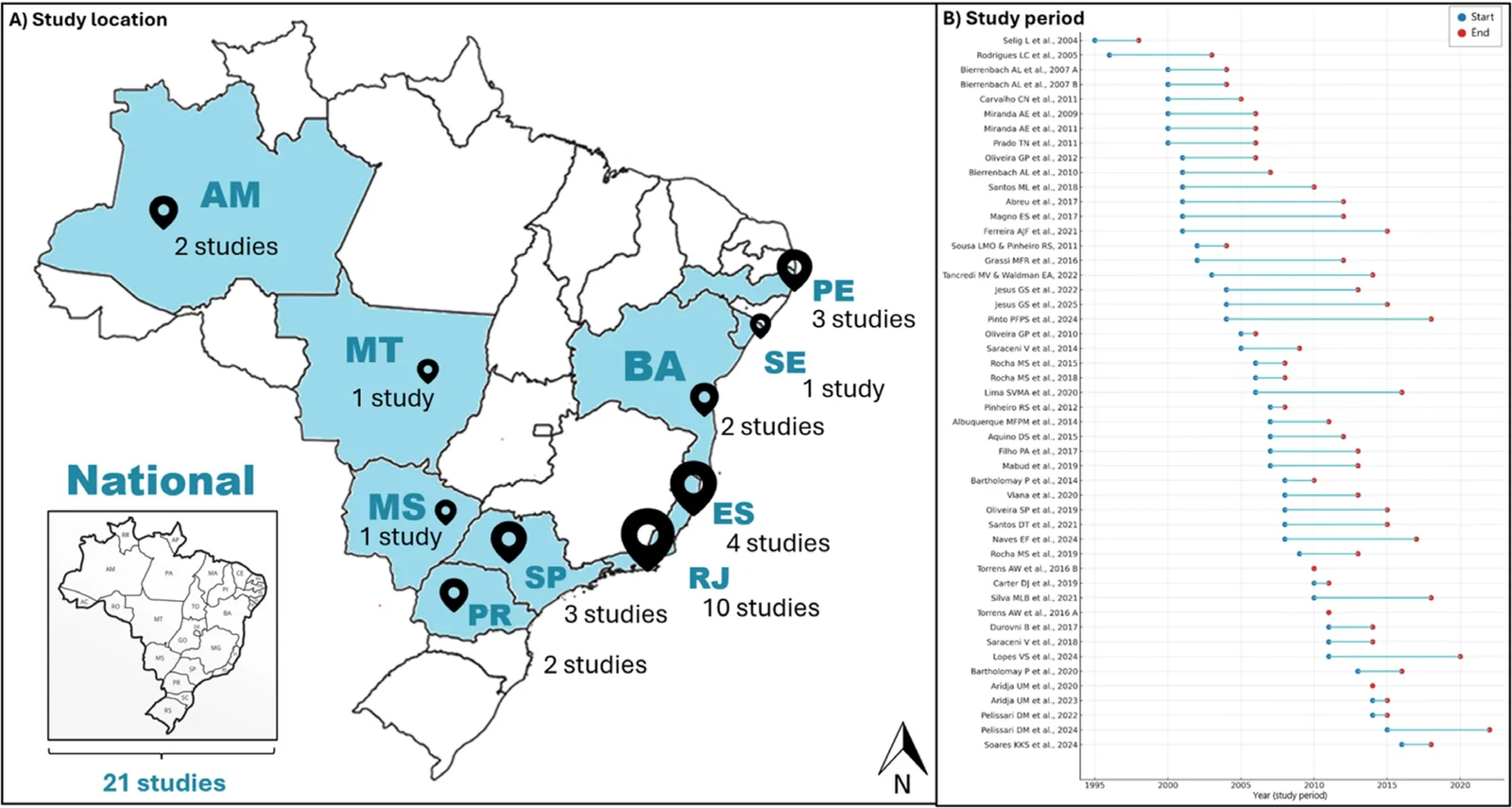
Information on locations* and periods of the included studies.

Note: a) Study location b) Analyzed period.

In addition, 21 articles (42.9%) had Brazil as a whole as the study location, and the second most frequent study location was the city of Rio de Janeiro, with 11 articles (22.4%). The analyzed study periods varied, presenting a median of six years (minimum: one year; maximum: 15 years) of study in each analysis (Figure 3).

*Some studies were multicentric, in such a way that the sum of the study locations exceeds 49.

Of the 49 studies, the use of the tuberculosis module of SINAN (SINAN-TB) comprised the majority, being used in 48 articles (98%). The second most used HIS was SIM, present in 33 articles (67.3%). Other databases were also greatly used, but on a smaller degree, such as the Single Registry for Federal Government Social Programs (*Cadastro Único para Programas Sociais do Governo Federal* – CadÚnico), the Benefit Management System of the *Bolsa Família* Program (conditional cash transfer program), the Information System of Special Treatments for Tuberculosis (*Sistema de Informação de Tratamentos Especiais da Tuberculose* – Site-TB), the Laboratory Environment Management System (*Sistema Gerenciador de Ambiente Laboratorial* – GAL), the Laboratory Test Control System of CD4/CD8 and Viral Load (*Sistema de Controle de Exames Laboratoriais de CD4/CD8 e Carga Viral* – Siscel), the Logistic Medicines Control System (*Sistema de Controle Logístico de Medicamentos* – Siclom), the AIDS module of SINAN (SINAN-AIDS), among others (Supplementary Table 1).

Eight articles (16.3%) did not report the results of record linkage, that is, how many pairs were found. Among those who reported such information with transparency (n=42; 85.7%), the amount of data used varied, ranging from 411 tuberculosis cases to 1,090,375 notifications. Other databases, such as CadÚnico, have used more than 130 million records in the linkage process. The number of records used in the studies ranged from 25 to 168,804, with a median of 1,008.5 (Q1 321.8; Q3 4,447.0) and a mean of 13,551.4 (Standard deviation [SD] 37,549.2); whereas the percentage of linked pairs ranged between 0.1 and 97.3%, with a median of 31% (Q1 12.5%; Q3 60.8%) and mean of 38.5% (SD 29.8). Manual review of the pairs was used in 31 articles (63.3%) (Supplementary Table 1).

## DISCUSSION

In this review, we identified 49 articles that used record linkage techniques in studies on tuberculosis in Brazil. We predominantly found publications in national journals; studies of national scope or carried out in Rio de Janeiro; and analyses using SINAN-TB, SIM, and OpenRecLink. Together, the findings indicate that record linkage has been mainly used to describe epidemiological scenarios, investigate associated factors, and identify underreporting.

The application of record linkage in Brazil faces challenges due to the quality of HIS data (filling errors, lack of standardization, and incomplete fields)^7^. The approaches are divided into deterministic and probabilistic. Deterministic approaches require exact correspondence (for example, full name, date of birth, or CPF number) and are fast, with a low rate of false positives, but not flexible in the face of inconsistencies, frequent in Brazilian health systems^14^. In turn, the probabilistic approaches use similarity algorithms to combine attributes with small discrepancies, better dealing with variations in spelling and partial fields and increasing the sensitivity of record linkage; yet, the accuracy of both approaches can be compromised by low-quality data^14,15^.

Technical and operational challenges persist in the implementation of record linkage, especially in contexts with extensive databases, incomplete fields, and the need for manual review. These factors may influence the operational feasibility of record linkage and the consistency of the analyses carried out^10,16,17^.

The increase in specificity and sensitivity of record linkage processes allows health systems to identify tuberculosis transmission patterns and evaluate the effectiveness of public policies for disease control^8,18–20^. The use of computational tools for record linkage, such as RecLink^21^, and programming languages, such as R^22,23^ and Python^13,24^, requires technical training that is not always available in a homogeneous way among public health professionals.

RecLink was the most widely used software in this review, especially for its accessibility and user-friendly interface without programming experience. R- and Python-based solutions have also been mentioned in the included studies, especially in recent years, suggesting diversification of the employed tools^25,26^. Considering that, in this review, we did not formally compare software performance, these findings should be interpreted as a description of the observed panorama, and not as evidence of superiority of one tool over another.

Regarding the geographic scope, authors of many studies used national data, but no specific studies were identified for six states in the North, six in the Northeast, two in the Midwest, in addition to the states of Minas Gerais (Southeast), Rio Grande do Sul and Santa Catarina (South). Although record linkage has the potential to guide local decisions, its use remains concentrated at the national level or in the capitals^10,11,18–20,27–41^.

The percentage of data linked between databases widely varied, from 0.1^42^ to 97.3%^43^. Ten articles did not objectively inform this indicator^17–19,30,34,35,38,44–46^, which limits the evaluation of reproducibility and transparency of the employed procedures. Low percentages may reflect very extensive databases, more restrictive linkage criteria, or specific analytical objectives, while higher percentages tend to be found on databases with greater proximity of purpose or greater compatibility between identifiers^9,20,29,31,42,47–49^. In theory, all deaths from tuberculosis in the SIM should correspond to records in SINAN; in practice, this is not confirmed, motivating the active search for deaths from tuberculosis not registered in SINAN^39,50–52^.

Furthermore, among the three states with the highest occurrence of new cases of tuberculosis between 2021 and 2024 (São Paulo, Rio de Janeiro, and Pernambuco), all presented studies with record linkage—this may be indicative that tuberculosis surveillance in these states has integration with academia and stimulates the production of scientific knowledge that contributes to its control. However, it is worth highlighting that for other states with high incidence, such as Pará and Rio Grande do Sul, we did not identified studies, which deserves attention.

Currently, there is the proposal to implement the *e-SUS Linha da Vida* [Life Line] project—which seeks interoperability between the different HIS and allows the monitoring of users of the Brazilian Unified Health System (SUS) from birth to death^53^. In addition, there are positive perspectives for the improvement and facilitation of record linkage processes. As of 2025, the Ministry of Health started issuing the National Health Card (SUS Card) based on the CPF number, which may favor the standardization of identifiers between databases and, consequently, support implementing linkage routines^54^.

As long as SUS does not implement both proposals, the need for and dependence on record linkage techniques for integrated monitoring will continue to be challenges. Without significant structural and technological advances, monitoring hospitalizations, deaths, cases of tuberculosis, and co-infections with other diseases will continue to depend on these techniques and have limited impact on the epidemiological surveillance of tuberculosis.

With regard to the contribution to tuberculosis surveillance in Brazil, the reviewed articles highlighted the potential of record linkage to qualify the notification process, identify vulnerable populations—such as persons deprived of liberty—and evaluate the effectiveness of intersectoral actions^8,11,33,43,55^. Authors of national studies have shown that the use of record linkage can be feasible on a large scale, contributing to structural analysis and evidence-based public policies^8,19^.

According to the results, record linkage has been used for various purposes, such as identifying underreporting^39,50,52,56–58^, analyzing data quality^10,11,16,17,32,36,59^, describing epidemiological scenarios^31,33,47,60–63^, investigating co-infections and comorbidities^9,45,48,49,64^, assessing public policies—such as social programs or health care strategies—^8,18–20,30,34,35^ and analyzing factors associated with the disease^27,29,41,43,44,65–68^.

The most frequently employed HIS were SINAN-TB and SIM, whose linkage enables monitoring critical outcomes (such as deaths) and clinical events related to tuberculosis^40,41,46,61,69^. Moreover, we evidenced the integration of data from SINAN-TB, SINAN-AIDS, SIM, and GAL, allowing for the detection of underreported cases of tuberculosis and deaths not properly registered and providing more accurate estimates of the burden of the disease in the country.

This integration of databases showed significant gaps in mortality records, exposing failures in the surveillance of deaths from tuberculosis^46,69^. In addition, the use of these techniques allowed identifying inconsistencies in SINAN-TB records^16,59^, expanding the analytical potential of epidemiological information and strengthening the formulation of public policies for disease control.

The studies highlighted limitations of coverage and quality of HIS, especially due to the absence of critical information. Secondary databases undergo updating of variables, which constitutes intrinsic limitation in the context of tuberculosis. Nevertheless, they offer important advantages, such as wide spatial and temporal coverage and availability for large-scale analyses. Considering the diversity of tools identified, the findings suggest that the field has incorporated different record linkage solutions over time. Authors of future investigations can explore, in a comparative way, the performance, usability, and operational requirements of these tools in different contexts.

The concentration of studies at the national level or in few subnational scenarios indicates a gap in the literature, especially for Federative Units with high TB burden and lower representation in the reviewed scientific production.

Another relevant finding was the variability in the way of reporting the linkage procedures and their results, which made it difficult to obtain standardized information—such as number of pairs found and manual review criteria—especially when a flowchart summarizing the linkage process was presented.

Altogether, as per the included studies, record linkage has played a relevant role in the qualification of records, in the identification of underreporting, and in the expansion of the analytical possibilities of tuberculosis surveillance in Brazil.

The findings of this review suggest that record linkage remains a useful and necessary strategy to integrate information from different systems and support epidemiological analyses on tuberculosis. However, its application still depends on the quality of the records, the availability of identifiers, and the transparency with which the studies report their procedures.

All in all, according to the literature review, record linkage has been widely used for various purposes in the surveillance of tuberculosis in Brazil. At the same time, challenges remain related to the quality of information systems, the methodological heterogeneity of studies, and the concentration of production in certain geographical and analytical contexts.

This review has some limitations. There was difficulty in collecting data on the number of observations used in the linkage processes and the records effectively paired, due to the lack of objectivity of some articles when reporting this information. Many articles presented confusing writing and inconsistent sample calculations, directly interfering in the collection of information. Furthermore, some record linkage processes had their retrieval percentage reduced after manual reviews or application of epidemiological criteria related to data quality such as date inconsistency or duplicate records.

In addition, no formal evaluation of methodological quality or risk of bias of the included studies was performed, as the objective of the review was to describe methodological characteristics of the studies, and not to synthesize effect estimates or epidemiological associations; moreover, we verified heterogeneity of the study designs, analytical objectives, and forms of reporting the linkage procedures in the included studies.
