# Supplementary material for: Record linkage in tuberculosis epidemiological surveillance in Brazil: systematic methodological literature review
Source: Rev Bras Epidemiol. 2026 Aug 24;29:e260045. doi: 10.1590/1980-549720260045 (PMC13506216; doi:10.1590/1980-549720260045)
Supplement: Supplementary file 1 [file 1980-5497-rbepid-29-e260045-suppl1-pt.pdf]

Material Suplementar

Tabela suplementar 1. Características gerais e principais resultados dos artigos incluídos.

| Citação: Título em inglês | Objetivo | Delineamento* | Local | Período | Software | Sistemas de informação em saúde | Tamanho amostral | Pares encontrados | % pareamento |
|---|---|---|---|---|---|---|---|---|---|
| **Abreu et. al., 2017**: Tuberculose e diabetes – relacionamento probabilístico da base de dados par ao estudo da associação entre as doenças (9) | Analisar coinfecção ou comorbidade | Estudo descritivo | Brasil (nacional) | 2001-2012 | Reclink | SINAN-TB; Hiperdia | SINAN-TB: 1.090.375 Diabetes: 1.246.137 | 7.287 | 0,70% |
| **Albuquerque MFPM et al., 2014:** Empirical treatment for TB in HIV: Lessons from a cohort study of people living with HIV treated in Recife, Brazil (69) | Analisar fatores associados / determinantes | Coorte prospectiva | Recife, Pernambuco, Brasil | 2007–2011 | Reclink | SIM; base de uma coorte clínica local | PLHIV: 2.382 (800 pacientes apresentavam tosse e foram incluídos para o pareamento) SIM: ND | 95 | 11,90% |
| **Aquino DS et al., 2015:** Factors associated with treatment for latent tuberculosis in persons | Analisar fatores associados / determinantes | Coorte prospectiva | Recife, Pernambuco, Brasil | 2007-2012 | Reclink | SINAN-TB; SIM | SINAN-TB: 2.341 SIM: ND | ND | ND |

| | | | | | | | | | |
|---|---|---|---|---|---|---|---|---|---|
| living with HIV/AIDS (47) | | | | | | | | | |
| **Aridja UM et al., 2020:** Tuberculosis cases with post-mortem notification in Brazil, 2014: a descriptive study based on surveillance data (38) | Descrever cenários epidemiológicos | Estudo descritivo | Brasil (nacional) | 2014 | Reclink | SINAN-TB; SIM | SINAN-TB: 67.966<br>SIM: 7.268 | 4.447 | 61,20% |
| **Aridja UM et al., 2023:** Factors associated with post-mortem notification of tuberculosis cases in Brazil, 2014 (32) | Analisar fatores associados / determinantes | Caso-controle | Brasil (nacional) | 2014–2015 | Reclink | SINAN-TB; SIM | SINAN-TB: ND<br>SIM: 7.268, mas apenas 7.150 seguiram para o pareamento | 4.447 | 62,20% |
| **Bartholomay P et al., 2014:** Improved quality of tuberculosis data using *Record* linkage (21) | Melhorar qualidade dos dados | Descritivo | Brasil (nacional) | 2008–2010 | Reclink | SINAN-TB; SIM | SINAN-TB: 170.432<br>SIM: 22.360 | 9.059 óbitos pareados (7.939 considerados válidos após critério de até 270 dias entre diagnóstico e óbito) | 40,50% |

| | | | | | | | | | |
|---|---|---|---|---|---|---|---|---|---|
| **Bartholomay P et al., 2020:** Gaps in drug-resistant tuberculosis surveillance: probabilistic database linkage in Brazil (31) | Melhorar qualidade dos dados | Descritivo (captura-recaptura) | Brasil (exceto São Paulo) | 2013–2016 | Reclink | SINAN-TB; SIM; GAL; SITE-TB | SITE-TB (2013 a 2016): 2.428<br>SINAN-TB (2014 a 2016): 1.917<br>Demais bases: ND | SITE-TB: 2238<br>SINAN-TB: 1440<br>SITE-TB + GAL: 802 | SITE-TB: 92,2%<br>SINAN-TB: 75,1%<br>SITE-TB + GAL: 33,0% |
| **Bierrenbach AL et al., 2007 A:** Tuberculosis incidence and cure rates, Brazil, 2000-2004 (43) | Descrever cenários epidemiológicos | Descritivo | Brasil (nacional) | 2000–2004 | Não informado | SINAN-TB | SINAN-TB: 362.942 | ND | ND |
| **Bierrenbach AL et al., 2007 B:** Impact on tuberculosis incidence rates of removal of repeat notification *Record*s (10) | Melhorar qualidade dos dados | Descritivo | Brasil (nacional) | 2000–2004 | Link Plus | SINAN-TB | SINAN-TB: 482.501 | 127.157 | 26,30% |
| **Bierrenbach AL et al., 2010:** Duplicates and misclassification of tuberculosis notification *Record*s in Brazil, 2001-2007 (11) | Melhorar qualidade dos dados | Descritivo | Brasil (nacional) | 2001–2007 | Link Plus | SINAN-TB | SINAN-TB: 651.366 | 147.590 | 22,70% |

| | | | | | | | | | |
|---|---|---|---|---|---|---|---|---|---|
| **Carter DJ et al., 2019:** The impact of a cash transfer programme on tuberculosis treatment success rate: a quasi-experimental study in Brazil (25) | Analisar efeito de políticas públicas / programas | Coorte retrospectiva | Brasil (nacional) | 2010–2011 | R | SINAN-TB; Cad-Único (Coorte de 100 milhões de brasileiros); folha de pagamento do Bolsa Família | SINAN-Cad-Único-BFP data set (N=180.046) | 16.760 | 9,30% |
| **Carvalho CN et al., 2011:** Underreporting of tuberculosis and AIDS comorbidity: an application of the linkage method (42) | Analisar subnotificação | Descritivo | Brasil (nacional) | 2000–2005 | Link Plus | SINAN-TB; SINAN-Aids; sistemas de informação em saúdeCEL/SICLOM | SINAN-TB: 517.977<br>SINAN-AIDS: 419.795<br>sistemas de informação em saúdeCEL/SICLOM: ND | 32.501 | 32,80% |
| **Durovni B et al., 2017:** The impact of the Brazilian Family Health Strategy and the conditional cash transfer on tuberculosis | Analisar efeito de políticas públicas / programas | Coorte retrospectiva | Rio de Janeiro, RJ, Brasil | 2011–2014 | Reclink | SINAN-TB; Cad-Único (Coorte de 100 milhões de brasileiros); folha de pagamento do Bolsa Família; Prontuários da Estratégia Saúde da Família | SINAN: 26.931, mas apenas 15.362 casos de TB seguiram para o pareamento<br>Cad-Único: ND | 2.890 cobertos pelo Bolsa família | 18,80% |

| | | | | | | | | | |
|---|---|---|---|---|---|---|---|---|---|
| treatment outcomes in Rio de Janeiro: an individual-level analysis of secondary data (8) | | | | | | | | | |
| **Ferreira AJF et al., 2021:** Evaluating the health effect of a Social Housing programme, Minha Casa Minha Vida, using the 100 million Brazilian Cohort: a natural experiment study protocol (39) | Analisar efeito de políticas públicas / programas | Coorte retrospectiva | Brasil (nacional) | 2001–2015 | Não informado | SINAN-TB; SINAN-Hanseníase; SIM; Cad-Único (Coorte de 100 milhões de brasileiros) | ND | ND | ND |
| **Filho PA et al., 2017:** Socio-spatial inequalities related to tuberculosis in the city of Itaboraí, Rio de Janeiro (62) | Descrever cenários epidemiológicos | Ecológico | Itaboraí, Rio de Janeiro, Brasil | 2007–2013 | Reclink | SINAN-TB; SIM; SITE-TB | SINAN-TB: 1.182. mas apenas 943 seguiram para o pareamento<br>SIM: 79<br>SITE-TB: ND | 25 | 31,60% |
| **Grassi MFR et al., 2016:** Tuberculosis incidence in a cohort of | Descrever cenários epidemiológicos | Coorte retrospectiva | Salvador, Bahia, Brasil | 2002–2012 | SQL | SINAN-TB; Base de dados do CHTLV (Centro de | SINAN: 74.454<br>CHTLV: 6.495 | 73 | 1,10% |

| | | | | | | | | | |
|---|---|---|---|---|---|---|---|---|---|
| individuals infected with human T-lymphotropic virus type 1 (HTLV-1) in Salvador, Brazil (49) | | | | | | Referência para HTLV) | | | |
| **Jesus GS et al., 2022:** The effect of primary health care on tuberculosis in a nationwide cohort of 7,3 million Brazilian people: a quasi-experimental study (40) | Analisar efeito de políticas públicas / programas | Coorte retrospectiva | Brasil (nacional) | 2004–2013 | CIDACS-RL | SINAN-TB; SIM; Cad-Único (Coorte de 100 milhões de brasileiros) | Cad-Único: 114.008.317<br>SINAN-TB: 266.556 | ND | ND |
| **Jesus GS et al., 2025:** Effects of conditional cash transfers on tuberculosis incidence and mortality according to race, ethnicity and socioeconomic factors in the 100 million Brazilian Cohort (24) | Analisar efeito de políticas públicas / programas | Coorte retrospectiva | Brasil (nacional) | 2004–2015 | CIDACS-RL | SINAN-TB; SIM; Cad-Único (Coorte de 100 milhões de brasileiros) | Cad-Único: 131.767.381;<br>SINAN-TB: 547.858<br>SIM: ND | ND | ND |
| **Lima SVMA et al., 2020:** | Melhorar qualidade dos dados | Ecológico | Sergipe, Brasil | 2006–2016 | Excel | SINAN-TB; SIM | SINAN-TB: 7.873, mas apenas 7.643 seguiram para o pareamento | 293 | 60,70% |

| | | | | | | | | | |
|---|---|---|---|---|---|---|---|---|---|
| Quality of tuberculosis information systems after *Record* linkage (61) | | | | | | | (após remoção de duplicidades);<br><br>SIM: 483 | | |
| **Lopes VS et al., 2024:**<br>Factors associated with deaths by tuberculosis in the state of Mato Grosso, 2011-2020: retrospective cohort study (67) | Analisar fatores associados / determinantes | Coorte retrospectiva | Mato Grosso, Brasil | 2011–2020 | Reclink | SINAN-TB; SIM | SINAN-TB: 12.331<br><br>SIM: 717 | 218 | 30,40% |
| **Mabud et. Al., 2019**:<br>Evaluating strategies for control of tuberculosis in prisons and prevention of spillover into communities: An observational and modeling study from Brazil (57) | Analisar intervenção ou tratamento / eficácia terapêutica | Coorte retrospectiva | Mato Grosso do Sul | 2007-2013 | OpenRefine | SINAN-TB; Sistema Prisional | SINAN-TB: 5.643<br><br>Sistema Prisional: 42.925 | 1.325 | 23,50% |
| **Magno ES et al., 2017:** | Analisar subnotificação | Transversal analítico | Amazonas, Brasil | 2001–2012 | Reclink | SINAN-TB; SINAN-AIDS; SIM; | SINAN-TB: 30.860 | SINAN-TB + SINAN-Aids: 1.469 | SINAN-TB + SINAN-Aids: 21,7% |

| | | | | | | | | | |
|---|---|---|---|---|---|---|---|---|---|
| Factors associated with TB/HIV coinfection: evidence from notification data in the State of Amazonas, Brazil, 2001-2012 (59) | | | | | | | SINAN-AIDS: 6.753<br><br>SIM: ND | SIM + resultado do pareamento anteriores pós critérios de exclusão: 426 | SIM + resultado do pareamento anteriores pós critérios de exclusão: 31,1% |
| **Miranda AE et al., 2009:**<br><br>*Tuberculosis and AIDS Co-Morbidity in Brazil:* linkage of the Tuberculosis and AIDS Databases (51) | Analisar coinfecção ou comorbidade | Descritivo | Espírito Santo, Brasil | 2000–2006 | Reclink | SINAN-TB; SINAN-AIDS; SIM; sistemas de informação em saúdeCEL/SICLOM; | Casos de Aids (Todas as 4 bases de dados - exceto SIM): 3.523<br><br>SINAN-TB: 9.958<br><br>Demais bases: ND | 430 | 12,20% |
| **Miranda AE et al., 2010:**<br>Tuberculosis and AIDS Co-Morbidity in Children: linkage of | Analisar coinfecção ou comorbidade | Descritivo | Espírito Santo, Brasil | 2000–2006 | Reclink | SINAN-TB; SINAN-AIDS; SIM; sistemas de informação em | SINAN-TB: 411<br><br>Demais bases: ND | 27 | 6,60% |

| | | | | | | | | | |
|---|---|---|---|---|---|---|---|---|---|
| Databases from Espírito Santo State, Brazil (65) | | | | | | saúdeCEL/SICLOM; | | | |
| **Naves EF et al., 2024:**<br>Factors associated with death due to tuberculosis/HIV co-infection in the prison system (66) | Analisar fatores associados / determinantes | Coorte retrospectiva | São Paulo, Brasil | 2008–2017 | R | SINAN-TB; SIM | SINAN-TB: 2.246<br>SINAN-HIV: 3.145<br>SIM: ND | 312 | 13,80% |
| **Oliveira GP et al., 2010:**<br>Linkage between SIM and SINAN for the improvement of the data quality in the tuberculosis information system: the Brazilian experience (41) | Melhorar qualidade dos dados | Descritivo | Brasil (nacional) | 2005–2006 | Reclink | SINAN-TB; SIM | SINAN-TB: 177.762<br>SIM: 6.924 | 3.400 | 49,10% |
| **Oliveira GP et al., 2012:**<br>Mortality information system for identifying underreported cases of tuberculosis in Brazil (44) | Analisar subnotificação | Descritivo | Brasil (nacional) | 2001–2006 | Reclink | SINAN-TB; SIM | SINAN-TB: 547.589<br>SIM: 6.924 | 4.197 | 60,60% |
| **Oliveira SP et al., 2019:** | Descrever cenários | Transversal | Paraná, Brasil | 2008–2015 | Reclink | SINAN-TB; SIM | SINAN-TB: 44.884 | 829 | 87,80% |

| | | | | | | | | | |
|---|---|---|---|---|---|---|---|---|---|
| Early death by tuberculosis as the underlying cause in a state of Southern Brazil: Profile, comorbidities and associated vulnerabilities (17) | epidemiológicos | | | | | | SIM: 944 | | |
| **Pelissari DM et al., 2022:** Environmental factors associated with the time to tuberculosis diagnosis in prisoners in São Paulo, Brazil (46) | Analisar fatores associados / determinantes | Coorte retrospectiva | São Paulo, Brasil | 2014–2015 | Não informado | SINAN-TB; Sistema prisional; Inquérito de condições ambientais | Sistema prisional: 4.112<br><br>SINAN-SINAN-TB: ND | 3.999 | 97,30% |
| **Pelissari DM et al., 2024:** Time until tuberculosis recurrence and associated factors in Brazil: a population based retrospective cohort study using a linked database (34) | Analisar fatores associados / determinantes | Coorte retrospectiva | Brasil (nacional) | 2015–2022 | Reclink | SINAN-TB | SINAN-TB: 69.868 | 3.253 pares (recorrência) | 6,50% |
| **Pinheiro RS et al., 2012:** | Analisar subnotificação | Transversal | Município do Rio de | 2007–2008 | Reclink | SINAN-TB; SIM; LPATB/LRLAB | SINAN-TB: 1.090 | SINAN-TB + SIM: 69 | SINAN-TB + SIM: 55,2% |

| | | | | | | | | | |
|---|---|---|---|---|---|---|---|---|---|
| Underreporting of tuberculosis in the Information System on Notifiable Diseases (SINAN): primary default and case detection from additional data sources using probabilistic *Record* linkage (54) | | | Janeiro, Brasil | | | | SIM: 125<br>LPATB/LRLAB: 1.149 | SINAN-TB + LPATB: 1.091 | SINAN-TB + LPATB: 94,7% |
| **Pinto PFPS et al., 2024:** Incidence and risk factors of tuberculosis among 420 854 household contacts of patients with tuberculosis in the 100 million Brazilian Cohort (2004-18): a cohort study (33) | Descrever cenários epidemiológicos | Coorte retrospectiva | Brasil (nacional) | 2004–2018 | CIDACS-RL | SINAN-TB; SIM; Cad-Único (Coorte de 100 milhões de brasileiros) | Cad-Único: 84 739.052<br>SINAN-TB: 1.314.626<br>Contatos: 420.854 de 137.131 pacientes índice<br>SIM: ND | 168.804 | 12,80% |
| **Prado TN et al., 2011:** | Descrever cenários | Estudo descritivo | | 2000–2006 | Reclink | SINAN-TB; SINAN-AIDS | SINAN-TB: 9.958 | 437 | 11,50% |

| | | | | | | | | | |
|---|---|---|---|---|---|---|---|---|---|
| Epidemiological profile of adult patients with tuberculosis and AIDS in the state of Espírito Santo, Brazil: Cross-referencing tuberculosis and AIDS databases (63) | epidemiológicos | retrospectivo com relacionamento de dados | Espírito Santo, Brasil | | | | SINAN-AIDS: 3.795 | | |
| **Rocha MS et al., 2015:** What are the causes of death of patients with tuberculosis: multiple causes of death in a cohort of cases and a research proposal of presumed causes (19) | Descrever cenários epidemiológicos | Coorte retrospectiva | Município do Rio de Janeiro, Brasil | 2006–2008 | Reclink | SINAN-TB; SIM | SINAN-TB: 6.370 SIM: 825 | ND | ND |
| **Rocha MS et al., 2018:** Effect of inpatient and outpatient care on treatment outcome in tuberculosis - a cohort study (68) | Analisar fatores associados / determinantes | Coorte retrospectiva | Município do Rio de Janeiro, Brasil | 2006–2008 | Reclink | SINAN-TB; SIM | SINAN-TB: 6.986, mas apenas 6.344 seguiram para o pareamento SIM: 118.000 | 799 | 12,60% |
| **Rocha MS et al., 2019:** | | Descritivo | Município do Rio | 2009–2013 | Reclink | SINAN-TB; SINAN-Aids; | SINAN-TB: 37.531 | ND | ND |

| | | | | | | | | | |
|---|---|---|---|---|---|---|---|---|---|
| Use of database linkage and scripting rules to upgrade variables in the SINAN-TB database (22) | Melhorar qualidade dos dados | | de Janeiro, Brasil | | | SIM; sistemas de informação em saúdeCEL/SICLOM | Demais bases: ND | | |
| **Rodrigues LC et al., 2005:** Effect of BCG revaccination on incidence of tuberculosis in school-aged children in Brazil: the BCG-REVAC cluster-randomised trial (45) | Analisar tratamento / eficácia terapêutica | Ensaio clínico randomizado por clusters | Salvador (BA) e Manaus (AM), Brasil | 1996–2003 | Não informado | SINAN-TB + Base do estudo | Base do estudo: 348.083<br><br>SINAN-TB: ND | 279 | 0,10% |
| **Santos DT et al., 2021:** Survival time among patients who were diagnosed with tuberculosis, the precocious deaths and associated factors in southern Brazil (16) | Analisar fatores associados / determinantes | Coorte retrospectiva | Curitiba, Paraná, Brasil | 2008–2015 | Excel, Rstudio | SINAN-TB; SIM; SITE-TB | SINAN: ND<br><br>SIM: 205 | 179 | 87,30% |
| **Santos ML et al., 2018:**<br><br>Factors associated with underreporting of | Analisar subnotificação | Transversal | Pernambuco, Brasil | 2001–2010 | Reclink | SINAN-TB; SINAN-AIDS | SINAN-TB: 51.488, mas apenas 46.642 seguiram para o pareamento<br>SINAN-AIDS: 9.350, mas | 926 | 70,80% |

| | | | | | | | | | |
|---|---|---|---|---|---|---|---|---|---|
| tuberculosis based on data from SINAN Aids and SINAN TB (60) | | | | | | | apenas 1.307 seguiu para o pareamento | | |
| **Saraceni V et al., 2014:**<br>Prevalent Tuberculosis (TB) at HIV diagnosis in Rio de Janeiro, Brazil: The TB/HIV in Rio (THRio) Cohort (64) | Descrever cenários epidemiológicos | Coorte retrospectiva | Rio de Janeiro, Brasil | 2005–2009 | Não informado | SINAN-TB; SIM; THRio cohort | THRio: > 19.000<br>Demais bases: ND | ND | ND |
| **Saraceni V et al., 2018:**<br>Tuberculosis burden on AIDS in Brazil: A study using linked databases (36) | Descrever cenários epidemiológicos | Coorte retrospectiva | Brasil (nacional) | 2011–2014 | Reclink | SINAN-TB; SINAN-AIDS; SIM; sistemas de informação em saúdeCEL/SICLOM; | SINAN-AIDS: 245.913<br>Demais bases: ND | 15.543 | 6,30% |
| **Selig L et al., 2004:**<br>Deaths attributed to tuberculosis in the state of Rio de Janeiro (18) | Descrever cenários epidemiológicos | Descritivo | Rio de Janeiro, Brasil | 1995–1998 | Reclink | SINAN-TB; SIM | SINAN-TB: 16.567<br>SIM: 1.146 | 478 | 41,70% |
| **Silva MLB et al., 2021:**<br>Factors associated with underreporting of cases of multidrug-resistant tuberculosis in the state of Rio de Janeiro, | Analisar subnotificação | Coorte retrospectiva | Rio de Janeiro, Brasil | 2010–2018 | Reclink | SIM; GAL; SITE-TB | SITE-TB: 3.358<br>GAL: 1.047 | 879 | 84,00% |

| | | | | | | | | | |
|---|---|---|---|---|---|---|---|---|---|
| Brazil: probabilistic database linkage (53) | | | | | | | SITE-TB: ND | | |
| **Soares KKS et al., 2024:** Analysis of the Vulnerability Profile of tuberculosis co-infection in people living with HIV (50) | Analisar coinfecção ou comorbidade | Descritivo | Espírito Santo, Brasil | 2016–2018 | Excel | SINAN-TB; SINAN-HIV | SINAN-TB: 4.428<br>SINAN-AIDS: ND | 325 | 7,30% |
| **Sousa LMO & Pinheiro RS, 2011:** Unnotified deaths and hospital admissions for tuberculosis in the municipality of Rio de Janeiro (52) | Analisar subnotificação | Descritivo | Município do Rio de Janeiro, Brasil | 2002–2004 | Reclink | SINAN-TB; SIM; SIH-SUS | SIM: 542<br>SIH-SUS: 1.079<br>SINAN-TB: ND | SINAN-TB + SIM: 308<br>SINAN-TB + SIH-SUS: 841<br>SIM + SIH: 71 | SINAN-TB + SIM: 56,8%<br>SINAN-TB + SIH-SUS: 77,9% |
| **Tancredi MV et al., 2022:** Mortality and survival of tuberculosis coinfected patients living with AIDS in São | Analisar coinfecção ou comorbidade | Coorte retrospectiva | São Paulo, Brasil | 2003–2014 | Não informado | SINAN-AIDS; SIM; sistemas de informação em saúdeCEL/SICLOM | SINAN-AIDS: 35.515 registros incluídos após exclusões<br>SIM: 10.973 | ND | ND |

| | | | | | | | | | |
|---|---|---|---|---|---|---|---|---|---|
| Paulo, Brazil: a 12-year cohort study (48) | | | | | | | | | |
| **Torrens AW et al., 2016 A:** HIV testing, antiretroviral therapy, and treatment outcomes in new cases of tuberculosis in Brazil, 2011 (35) | Analisar efeito de políticas públicas / programas | Transversal e coorte retrospectiva | Brasil (nacional) | 2011 | Reclink | SINAN-TB; sistemas de informação em saúdeCEL/SICLOM | SINAN-TB: 73.741<br><br>sistemas de informação em saúdeCEL/SICLOM: ND | ND | ND |
| **Torrens AW et al., 2016 B:** Effectiveness of a conditional cash transfer programme on TB cure rate: a retrospective cohort study in Brazil (23) | Analisar efeito de políticas públicas / programas | Coorte retrospectiva | Brasil (nacional) | 2010 | Não informado | SINAN-TB; Cad-Único (Coorte de 100 milhões de brasileiros); folha de pagamento do Bolsa Família | SINAN-TB: 71.660<br><br>Cad-Único: ND | 17.133 | 23,80% |
| **Viana et. al., 2020:**<br><br>Factors associated with death in patients with tuberculosis in Brazil: Competing risks analysis (20) | Analisar fatores associados / determinantes | Coorte retrospectiva | Brasil (nacional) | 2008–2013 | STATA; R | SINAN-TB;<br><br>SIM | SINAN-TB: 283.508<br><br>SIM: 39.997 | 12.301 | 30,80% |

Nota: ND: Não Disponível/Não informado/ Não possível de calcular/Informação não está clara. sistemas de informação em saúde: Sistemas de Informação em Saúde. *O delineamento dos estudos foi apresentado conforme descrito pelos próprios autores.
