# Supplementary material for: Record linkage in tuberculosis epidemiological surveillance in Brazil: systematic methodological literature review
Source: Rev Bras Epidemiol. 2026 Aug 24;29:e260045. doi: 10.1590/1980-549720260045 (PMC13506216; doi:10.1590/1980-549720260045)
Supplement: Translation

# Translation: Vinculação de dados para vigilância epidemiológica da tuberculose no Brasil: revisão metodológica sistemática da literatura

**DOI:** 10.1590/1980-549720260045.2

**Authors:** Klauss K. S. Garcia, Amanda Amaral Abrahão, Henry Maia Peixoto, Elisabeth Carmen Duarte, Wildo Navegantes de Araújo, Walter Massa Ramalho

**Affiliations:** I London School of Hygiene and Tropical Medicine, Faculty of Epidemiology and Population Health, Department of Infectious Disease Epidemiology and International Health – Londres, Reino Unido.; II Universidade de Brasília, Núcleo de Medicina Tropical – Brasília (DF), Brasil.; III Universidade de Brasília, Faculdade de Ciências da Saúde – Brasília (DF), Brasil.; IV Universidade de Brasília, Faculdade de Ciências e Tecnologia em Saúde – Brasília (DF), Brasil.

**Keywords:** Record linkage, Sistemas de Informação em Saúde, Tuberculose, Vigilância em Saúde Pública, Revisão Sistemática

## Abstract

**Objetivo:**

Descrever a literatura sobre as características metodológicas e o uso de técnicas de vinculação de dados aplicadas à vigilância da tuberculose no Brasil.

**Métodos:**

Revisão metodológica sistemática (PROSPERO: CRD42024623969) conduzida segundo as diretrizes *Preferred Reporting Items for Systematic Reviews and Meta-Analyses* (PRISMA) 2020. As buscas foram realizadas nas bases de dados *PubMed*, Embase, *Scopus*, Biblioteca Virtual em Saúde (BVS), *Scientific Electronic Library Online (SciELO)* e Literatura Latino-Americana e do Caribe em Ciências da Saúde (LILACS) em 5 de fevereiro de 2025. Incluíram-se artigos científicos conduzidos no Brasil que aplicaram vinculação de dados entre sistemas de informação em saúde para fins de vigilância epidemiológica. Excluíram-se literatura cinzenta e estudos sem foco em tuberculose, sem aplicação de vinculação e realizados fora do Brasil. A síntese foi descritiva, reportando frequências, proporções e medidas de tendência central. Não foi realizada avaliação de risco de viés ou qualidade metodológica, devido à heterogeneidade dos delineamentos e objetivos, também porque o objetivo desta revisão foi descrever características metodológicas dos estudos e não sintetizar estimativas de efeito ou associações epidemiológicas.

**Resultados:**

A busca retornou 1.549 registros; 49 estudos foram incluídos. A maioria utilizou o Sistema de Informação de Agravos de Notificação (Sinan-TB) (98%) e Sistema de Informação sobre Mortalidade (SIM) (67,3%); o software mais empregado foi o *OpenRecLink* (57,1%). As variáveis-chave mais comuns foram "nome", "data de nascimento" e "nome da mãe". O percentual de vinculação variou de 0,1% a 97,3%. Os objetivos mais frequentes foram a descrição de cenários epidemiológicos (24,5%), análise de fatores associados (20,4%) e investigação de subnotificação (16,3%). Predominaram delineamentos de coorte (49%) e descritivos (34,7%).

**Conclusões:**

A vinculação de dados integra bases complementares e pode fortalecer a vigilância da tuberculose. Os achados sugerem potencial utilidade da vinculação de dados para apoiar análises epidemiológicas, qualificar registros e subsidiar ações de vigilância.

## INTRDUÇÃO

A tuberculose é uma das principais doenças infecciosas no mundo e no Brasil, apresentando alta incidência, especialmente em regiões com desigualdades socioeconômicas acentuadas^1^. O Brasil está entre as nações com maior carga de tuberculose no mundo^1^, com taxas de incidência acima de 37 casos para cada 100 mil habitantes e mais de 80 mil casos novos de tuberculose todos os anos desde a pandemia de covid-19^2^.

Para eliminar a tuberculose, o Brasil adotou uma série de medidas previstas no plano EndTB, que visa eliminar a doença no país até 2035. Este plano está alinhado aos Objetivos de Desenvolvimento Sustentável (ODS), que, em sua Meta 3.3, propõe a eliminação de epidemias de doenças infecciosas e parasitárias, incluindo a tuberculose, até 2030^1,3,4^.

Entre as várias questões que influenciam o processo de eliminação da tuberculose no Brasil, podem ser destacados os desafios de se utilizar Sistemas de Informação em Saúde (SIS), como o Sistema de Informação de Agravos de Notificação (SINAN) e o Sistema de Informação sobre Mortalidade (SIM), que não possuem interoperabilidade entre si, dificultando o monitoramento integrado de casos de tuberculose e óbitos, por exemplo^5–7^.

Para superar esta limitação tecnológica, utilizam-se técnicas de vinculação de dados (*record linkage*) para o relacionamento dos SIS. Esse processo consiste em identificar e combinar registros que correspondem a um mesmo indivíduo em bases de dados distintas, mesmo quando não existe um identificador único em comum, que poderia ser o Cadastro de Pessoa Física (CPF) ou o Cartão Nacional de Saúde (Cartão SUS)^7^.

No contexto da vigilância epidemiológica da tuberculose, a vinculação permite uma visão mais completa do histórico clínico dos pacientes e dos desfechos^8,9^. Além disso, essas técnicas ajudam a detectar e eliminar duplicações de registros (técnica de deduplicação), consolidando notificações não repetidas^10,11^. Em geral, o processo envolve etapas prévias de preparação dos dados, como limpeza, padronização e, em alguns estudos, blocagem, com o objetivo de tornar a vinculação mais viável e eficiente^12^.

Considerando que o uso de técnicas de vinculação de dados é amplamente difundido, sobretudo no âmbito da vigilância da tuberculose, esta revisão sistemática tem como objetivo descrever as características metodológicas e o uso de técnicas de vinculação aplicadas a estudos sobre tuberculose no Brasil e, com base nesses achados, discutir como essas técnicas são e podem ser utilizadas como ferramenta de apoio à vigilância epidemiológica da tuberculose.

## MÉTODOS

Esta revisão metodológica sistemática foi registrada na plataforma *International Prospective Register of Systematic Reviews* (PROSPERO) sob o identificador CRD42024623969 e elaborada conforme as diretrizes *Preferred Reporting Items for Systematic Reviews and Meta-Analyses* (PRISMA) 2020.

### Contexto

O estudo está inserido no contexto brasileiro sob orientação da seguinte pergunta de pesquisa: "Como as técnicas de vinculação de dados têm sido utilizadas em estudos epidemiológicos sobre a tuberculose no Brasil?", tendo como foco principal a aplicação de técnicas de vinculação de dados provenientes dos SIS utilizados pela vigilância epidemiológica da tuberculose no país.

### Desenho do estudo

Trata-se de uma revisão metodológica sistemática da literatura com o objetivo de descrever características metodológicas dos estudos que aplicaram técnicas de vinculação de dados para a vigilância da tuberculose no Brasil. Além disso, buscou-se avaliar os contextos de aplicação dessas técnicas, os desafios enfrentados e as oportunidades resultantes do seu uso no âmbito dos SIS.

### Estratégias de busca e bases de dados

A busca final foi realizada em 5 de fevereiro de 2025 em 6 bases de dados eletrônicas: *PubMed*, Embase, *Scopus*, Biblioteca Virtual em Saúde (BVS), *Scientific Electronic Library Online* (*SciELO*) e Literatura Latino-Americana e do Caribe em Ciências da Saúde (LILACS). As estratégias foram adaptadas para cada base e combinaram descritores controlados e termos livres relacionados a vinculação de dados, tuberculose e Brasil, com uso explícito de operadores booleanos. A Tabela 1 apresenta, por base, as expressões empregadas e sua estrutura de combinação.

**Tabela 1:** Estratégias de busca utilizadas.

Base	Estratégia
*PubMed*	(("linkage" OR "linked" OR "Medical *Record* linkage "[Mesh] OR "Medical *Record* linkage " OR "Health *Record* linkage " OR "*Record* linkage " OR "Data linkage " OR "Centre for Data and Knowledge Integration for Health" OR "CIDACS") AND (Brazil OR Brazilian) AND (Tuberculosis))
Embase	‘tuberculosis’ AND [embase]/lim AND (‘brazil’ OR ‘brazilian’) AND [embase]/lim AND (linkage OR ‘linked’ OR ‘medical *Record* linkage /exp OR ‘medical *Record* linkage OR ‘health *Record* linkage OR ‘*Record* linkage OR ‘data linkage OR ‘centre for data and knowledge integration for health’ OR ‘cidacs’) AND [embase]/lim AND ‘article’/it
*Scopus*	ALL("linkage " OR "linked" OR "medical *Record* linkage " OR "health *Record* linkage " OR "*Record* linkage " OR "data linkage " OR "centre for data and knowledge integration for health" OR "CIDACS") AND TITLE-ABS-KEY(tuberculosis) AND AFFILCOUNTRY("Brazil") AND DOCTYPE("ar") AND NOT (SUBJAREA("IMMU") OR SUBJAREA("BIOC") OR SUBJAREA("AGRI"))
BVS	("vinculação" OR "vinculado" OR "vinculação de registros médicos" OR "vinculação de dados" OR "ligação de registros médicos" OR "CIDACS" OR "vinculación" OR "vinculado" OR "vinculación de registros médicos" OR "vinculación de datos" OR "enlace de registros médicos" OR " linkage " OR "linked" OR "medical *Record* linkage " OR "health *Record* linkage " OR "*Record* linkage " OR "data linkage " OR "Centre for Data and Knowledge Integration for Health") AND ("Brasil" OR "brasileiro" OR "brasileira" OR "brasileiros" OR "brasileiras" OR "Brasil" OR "brasileño" OR "brasileña" OR "brasileños" OR "brasileñas" OR "Brazil" OR "Brazilian") AND ("tuberculose" OR "tuberculosis")
*SciELO*	("vinculação" OR "vinculado" OR "vinculação de registros médicos" OR "vinculação de dados" OR "ligação de registros médicos" OR "CIDACS" OR "vinculación" OR "vinculado" OR "vinculación de registros médicos" OR "vinculación de datos" OR "enlace de registros médicos" OR " linkage " OR "linked" OR "medical *Record* linkage " OR "health *Record* linkage " OR "*Record* linkage " OR "data linkage " OR "Centre for Data and Knowledge Integration for Health") AND ("Brasil" OR "brasileiro" OR "brasileira" OR "brasileiros" OR "brasileiras" OR "Brasil" OR "brasileño" OR "brasileña" OR "brasileños" OR "brasileñas" OR "Brazil" OR "Brazilian") AND ("tuberculose" OR "tuberculosis" OR "tuberculose" OR "tuberculose")
LILACS	("vinculação" OR "vinculado" OR "vinculação de registros médicos" OR "vinculação de dados" OR "ligação de registros médicos" OR "CIDACS" OR "vinculación" OR "vinculado" OR "vinculación de registros médicos" OR "vinculación de datos" OR "enlace de registros médicos" OR " linkage " OR "linked" OR "medical *Record* linkage " OR "health *Record* linkage " OR "*Record* linkage " OR "data linkage " OR "Centre for Data and Knowledge Integration for Health") AND ("Brasil" OR "brasileiro" OR "brasileira" OR "brasileiros" OR "brasileiras" OR "Brasil" OR "brasileño" OR "brasileña" OR "brasileños" OR "brasileñas" OR "Brazil" OR "Brazilian") AND ("tuberculose" OR "tuberculosis")

### Local e população

O estudo centra-se em pesquisas realizadas no contexto brasileiro que empregaram técnicas de vinculação de dados aplicadas à vigilância epidemiológica da tuberculose, sem restrição temporal, de local ou população.

### Critérios de inclusão e exclusão

Incluíram-se artigos científicos que abordaram o uso de técnicas de vinculação de dados em investigações epidemiológicas sobre tuberculose no Brasil. Foram considerados elegíveis estudos que utilizaram dados provenientes de sistemas de informação em saúde brasileiros e que implementaram procedimentos de vinculação de registros.

Por sua vez, foram excluídos estudos cujo foco principal não fosse a tuberculose; realizados fora do Brasil; sem aplicação direta de vinculação de dados para fins de vigilância epidemiológica; ou que não estivessem em formato de artigo científico, como resumos de congresso, relatórios técnicos e documentos institucionais.

### Processo de seleção

As referências das seis bases foram exportadas para o Rayyan, consolidadas e deduplicadas. Dois pesquisadores triaram títulos e resumos segundo critérios predefinidos. Registros potencialmente elegíveis passaram à leitura na íntegra, com reaplicação dos critérios e remoção de duplicatas remanescentes. A seleção foi independente entre os dois pesquisadores.

Além da busca eletrônica, foi realizada busca manual nas listas de referências dos estudos incluídos, com o objetivo de identificar artigos potencialmente elegíveis não recuperados nas estratégias iniciais. Os estudos localizados foram avaliados segundo os mesmos critérios de elegibilidade e, quando pertinentes, incorporados ao conjunto final.

### Extração e análise de dados

A extração de dados foi realizada independentemente por dois pesquisadores para garantir consistência e qualidade. Variáveis extraídas: título, objetivo principal, ano e local de publicação, idioma, período do estudo, tipo de vinculação (probabilístico ou determinístico), uso de blocagem, variáveis-chave da vinculação de dados, SIS envolvidos e softwares utilizados. Para a tabulação e análise dos dados, foi utilizado o software Microsoft Excel, enquanto a linguagem Python^13^ foi empregada para a produção de gráficos e representações visuais dos resultados. Não foi realizada avaliação formal da qualidade metodológica ou do risco de viés, devido à heterogeneidade dos delineamentos, objetivos analíticos e formas de relato dos procedimentos de vinculação nos estudos incluídos.

#### Declaração de disponibilidade de dados:

Todos os dados utilizados nesta revisão foram extraídos de artigos científicos publicados e estão descritos na seção de métodos e na Tabela Suplementar 1. Não foram utilizados dados individuais, sigilosos ou não publicados.

## RESULTADOS

A pesquisa inicial resultou em 1.549 artigos, dos quais 678 foram identificados como duplicados e removidos. Após a leitura de títulos e resumos, 777 registros foram excluídos, restando 94 para leitura na íntegra. Após a avaliação dos textos completos, 47 artigos foram incluídos. Em seguida, dois estudos adicionais identificados por busca manual nas listas de referências foram incorporados, totalizando 49 estudos incluídos (Figura 1).

**Figura 1:** Fluxograma do processo de seleção dos artigos incluídos na revisão.

Os artigos foram publicados de 2004 a fevereiro de 2025, a média de publicações foi de 2,2 artigos por ano, e nos anos de 2006, 2008 e 2013 não foram identificadas publicações elegíveis. A revista com o maior número de publicações na temática foi a *Cadernos de Saúde Pública* (n=10; 20,4%), seguida pela *Revista Brasileira de Epidemiologia* (n=4; 8,2%) e a *Revista de Saúde Pública* (n=4; 8,2%) (Figura 2A). A maioria dos artigos foi publicada em revistas nacionais (n=29; 59,2%) (Figura 2B), mas majoritariamente em língua inglesa (n=28; 57,1%) (Figura 2C).

**Figura 2:** Principais características dos artigos incluídos.

Dos 49 artigos, 24 foram de estudos delineados como estudos de coorte (49%), 17 estudos descritivos (34,7%) e 5 estudos transversais (10,2%) (Figura 2D). Dos 49 artigos, o objetivo mais frequente foi descrever cenários epidemiológicos (n=12; 24,5%), seguido por analisar fatores associados a desfechos relacionados a tuberculose (n=10; 20,4%) e analisar subnotificação da tuberculose nos SIS (n=8; 16,3%) (Figura 2E). Adicionalmente, a vinculação foi utilizada também para analisar cenários de coinfecções e impactos de programas e políticas públicas na incidência da tuberculose (Figura 2E).

O software mais utilizado foi o *OpenRecLink* (versão 3) (n=28; 57,1%), seguido pelo R (n=4; 8,2%) e Cidacs-RL, Linkplus e Microsoft Excel (3 artigos para cada software; 6,1%), enquanto 8 artigos (16,3%) não relataram quais softwares utilizaram (Figura 2F). Observou-se que a utilização do *RecLink* se manteve constante ao longo dos anos e que, a partir de 2019, houve maior diversidade de softwares, incluindo R e os algoritmos do Cidacs-RL (Figura 2G).

As variáveis-chave mais frequentemente utilizadas foram a combinação de "nome do paciente" (n=39 artigos; 79,6%), "data de nascimento" (n=39 artigos; 79,6%) e "nome da mãe" (n=34 artigos; 69,4%), enquanto 10 artigos (20,4%) não relataram quais as variáveis-chave de vinculação utilizaram. Outros artigos, em menor frequência, utilizaram também "sexo" e informações de "endereço" como variáveis-chave.

Ainda, 21 artigos (42,9%) tiveram todo o Brasil como local de estudo, e o segundo local de estudo mais frequente foi a cidade do Rio de Janeiro, com 11 artigos (22,4%). Os períodos de estudos analisados variaram, apresentando uma mediana de 6 anos (mínimo: 1 ano; máximo: 15 anos) de estudo em cada análise (Figura 3).

**Figura 3:** Informações sobre locais* e períodos dos estudos incluídos.

Nota: a) Locais de estudos; b) Período analisado.

*Alguns estudos foram multicêntricos, então a soma dos locais de estudo excede 49.

Dos 49 estudos, a utilização do Sinan-TB foi majoritária, tendo sido utilizado em 48 artigos (98%). O segundo SIS mais utilizado foi o SIM, presente em 33 artigos (67,3%). Outras bases de dados também o foram, porém em menor escala, como o Cadastro Único para Programas Sociais do Governo Federal (CadÚnico), Sistema de Gestão de Benefícios do Programa Bolsa Família, Sistema de Informação de Tratamentos Especiais da Tuberculose (Site-TB), Sistema Gerenciador de Ambiente Laboratorial (GAL), Sistema de Controle de Exames Laboratoriais de CD4/CD8 e Carga Viral (Siscel), Sistema de Controle Logístico de Medicamentos (Siclom), Sinan-AIDS, entre outros (Tabela Suplementar 1).

Oito artigos (16,3%) não relataram os resultados da vinculação de dados, ou seja, quantos pares foram encontrados. Entre os que relataram tais informações com transparência (n=42; 85,7%), a quantidade de dados utilizada variou, abrangendo desde 411 casos de tuberculose até 1.090.375 notificações. Outras bases de dados, como a do CadÚnico, chegaram a utilizar mais de 130 milhões de registros no processo de vinculação. O número de registros utilizados nos estudos variou de 25 a 168.804, com mediana de 1.008,5 (Q1 321,8; Q3 4.447,0) e média de 13.551,4 (Desvio-Padrão [DP] 37.549,2); já o percentual de pares vinculados variou entre 0,1% e 97,3%, com mediana de 31% (Q1 12,5%; Q3 60,8%) e média de 38,5% (DP 29,8). Revisão manual dos pares foi empregada em 31 artigos (63,3%) (Tabela Suplementar 1).

## DISCUSSÃO

Esta revisão identificou 49 artigos que utilizaram técnicas de vinculação de dados em estudos sobre tuberculose no Brasil. Predominaram publicações em periódicos nacionais; estudos de abrangência nacional ou realizados no Rio de Janeiro; e análises com uso do Sinan-TB, do SIM e do *OpenRecLink*. Em conjunto, os achados indicam que a vinculação de dados tem sido empregada principalmente para descrever cenários epidemiológicos, investigar fatores associados e identificar subnotificação.

A aplicação da vinculação de dados no Brasil enfrenta desafios devido à qualidade dos dados dos SIS (erros de preenchimento, falta de padronização e campos incompletos)^7^. As abordagens dividem-se em determinísticas e probabilísticas. As determinísticas exigem correspondência exata (por exemplo, nome completo, data de nascimento ou CPF) e são rápidas, com baixa taxa de falsos positivos, porém pouco flexíveis diante de inconsistências, frequentes nos sistemas de saúde brasileiros^14^. Já as probabilísticas utilizam algoritmos de similaridade para combinar atributos com pequenas discrepâncias, lidando melhor com variações de grafia e campos parciais e elevando a sensibilidade da vinculação de dados; ainda assim, a acurácia de ambas as abordagens pode ser comprometida por dados de baixa qualidade^14,15^.

Persistem desafios técnicos e operacionais na implementação da vinculação de dados, especialmente em contextos com bases extensas, campos incompletos e necessidade de revisão manual. Esses fatores podem influenciar a viabilidade operacional da vinculação de dados e a consistência das análises produzidas^10,16,17^.

O aumento na especificidade e sensibilidade dos processos de vinculação de dados permite que os sistemas de saúde identifiquem padrões de transmissão da tuberculose e avaliem a efetividade de políticas públicas para o controle da doença^8,18–20^. A utilização de ferramentas computacionais para a vinculação de dados, como o *RecLink* ^21^ e linguagens de programação como R^22,23^ e Python^13,24^, requer capacitação técnica que nem sempre está disponível de forma homogênea entre os profissionais de saúde pública.

O *RecLink* foi o software mais utilizado nesta revisão, sobretudo por sua acessibilidade e interface amigável a usuários sem experiência em programação. Soluções baseadas em R e Python também apareceram nos estudos incluídos, especialmente nos anos mais recentes, sugerindo diversificação das ferramentas empregadas^25,26^. Como esta revisão não comparou formalmente o desempenho entre softwares, esses achados devem ser interpretados como descrição do panorama observado, e não como evidência de superioridade de uma ferramenta sobre outra.

Quanto ao âmbito geográfico, muitos estudos utilizaram dados nacionais, porém não foram identificados estudos específicos para 6 estados do Norte, 6 do Nordeste, 2 do Centro-Oeste, além de Minas Gerais (Sudeste), Rio Grande do Sul e Santa Catarina (Sul). Embora a vinculação de dados tenha potencial para orientar decisões locais, seu uso permanece concentrado no nível nacional ou em capitais^10,11,18–20,27–41^.

O percentual de dados vinculados entre bases variou amplamente, de 0,1^42^ a 97,3%^43^. Dez artigos não informaram objetivamente esse indicador^17–19,30,34,35,38,44–46^, o que limita a avaliação da reprodutibilidade e transparência dos procedimentos empregados. Percentuais baixos podem refletir bases muito extensas, critérios mais restritivos de vinculação ou objetivos analíticos específicos, enquanto percentuais mais elevados tendem a ocorrer em bases com maior proximidade de finalidade ou maior compatibilidade entre identificadores^9,20,29,31,42,47–49^. Em teoria, todos os óbitos por tuberculose do SIM deveriam corresponder a registros no Sinan; na prática, isso não se confirma, motivando a busca ativa de óbitos de tuberculose não registrados no Sinan^39,50–52^.

Ainda, entre os três estados com a maior ocorrência de casos novos de tuberculose entre 2021 e 2024 (São Paulo, Rio de Janeiro e Pernambuco), todos apresentaram estudos com vinculação de dados, o que pode ser indicativo de que a vigilância da tuberculose nesses estados possui integração com a academia e estimula a produção de conhecimento científico que contribua para o seu controle. No entanto, é importante destacar que outros estados com elevada incidência, como Pará e Rio Grande do Sul, não tiveram estudos identificados, o que merece atenção.

Atualmente existe a proposta de implementação do projeto e-SUS Linha da Vida – que busca interoperabilidade entre os diferentes SIS e permitir o acompanhamento do usuário do Sistema Único de Saúde (SUS) desde o nascimento até a morte^53^. Adicionalmente, há perspectivas positivas para o aprimoramento e a facilitação dos processos de vinculação de dados. A partir de 2025, o Ministério da Saúde passou a emitir o Cartão SUS com base no número de CPF, o que pode favorecer a padronização de identificadores entre bases de dados e, por consequência, apoiar a execução de rotinas de vinculação^54^.

Enquanto o SUS não implementar ambas as propostas, a necessidade e a dependência de técnicas de vinculação de dados para um monitoramento integrado continuarão sendo desafios. Sem avanços estruturais e tecnológicos significativos, monitorar internações, óbitos, casos de tuberculose e coinfecções com outras doenças continuará uma ação dependente dessas técnicas e com impacto limitado na vigilância epidemiológica da tuberculose.

No que tange à contribuição para a vigilância da tuberculose no Brasil, os artigos revisados evidenciaram o potencial da vinculação de dados para qualificar o processo de notificação, identificar populações vulneráveis – como pessoas privadas de liberdade – e avaliar a efetividade de ações intersetoriais^8,11,33,43,55^. Os estudos de abrangência nacional mostraram que o uso da vinculação de dados pode ser viável em larga escala, contribuindo para análises estruturantes e políticas públicas baseadas em evidências^8,19^.

Os resultados mostram que a vinculação de dados tem sido empregada com finalidades variadas, como identificar subnotificações^39,50,52,56–58^, analisar a qualidade dos dados^10,11,16,17,32,36,59^, descrever cenários epidemiológicos^31,33,47,60–63^, investigar coinfecções e comorbidades^9,45,48,49,64^, avaliar políticas públicas como programas sociais ou estratégias da atenção a saúde^8,18–20,30,34,35^ e analisar fatores associados à doença^27,29,41,43,44,65–68^.

Os SIS mais empregados foram o Sinan-TB e o SIM, cuja vinculação viabiliza monitorar desfechos críticos (como óbitos) e eventos clínicos relacionados à tuberculose^40,41,46,61,69^. Ainda, evidenciou-se a integração de dados provenientes do Sinan-TB, Sinan-AIDS, SIM e GAL, possibilitando a detecção de casos subnotificados de tuberculose e óbitos não registrados adequadamente e fornecendo estimativas mais acuradas da carga da doença no país.

Essa integração de bases de dados evidenciou lacunas significativas nos registros de mortalidade, expondo falhas na vigilância de óbitos por tuberculose^46,69^. Além disso, o uso dessas técnicas permitiu a identificação de inconsistências nos registros do Sinan-TB^16,59^, ampliando o potencial analítico das informações epidemiológicas e fortalecendo a formulação de políticas públicas de controle da doença.

Os estudos destacaram limitações de cobertura e qualidade dos SIS, sobretudo pela ausência de informações críticas. Bases secundárias passam por atualizações de variáveis, o que constitui limitação intrínseca no contexto da tuberculose. Ainda assim, oferecem vantagens importantes, como ampla cobertura espacial e temporal e disponibilidade para análises em grande escala. Considerando a diversidade de ferramentas identificadas, os achados sugerem que o campo tem incorporado diferentes soluções de vinculação de dados ao longo do tempo. Investigações futuras podem explorar de forma comparativa o desempenho, a usabilidade e as exigências operacionais dessas ferramentas em contextos distintos.

A concentração de estudos em nível nacional ou em poucos cenários subnacionais indica uma lacuna na literatura, especialmente para unidades federativas com elevada carga de tuberculose e menor representação na produção científica revisada.

Outro achado relevante foi a variabilidade na forma de relatar os procedimentos de vinculação e seus resultados, o que dificultou a extração padronizada de informações como número de pares encontrados e critérios de revisão manual, principalmente quando foi apresentado um fluxograma sumarizando o processo de vinculação.

Em conjunto, os estudos incluídos indicam que a vinculação de dados tem desempenhado papel relevante na qualificação de registros, na identificação de subnotificação e na ampliação das possibilidades analíticas da vigilância da tuberculose no Brasil.

Os achados desta revisão sugerem que a vinculação de dados permanece uma estratégia útil e necessária para integrar informações provenientes de diferentes sistemas e apoiar análises epidemiológicas sobre tuberculose. Contudo, sua aplicação ainda depende da qualidade dos registros, da disponibilidade de identificadores e da transparência com que os estudos reportam seus procedimentos.

Em síntese, a literatura revisada mostra que a vinculação de dados tem sido utilizada de forma ampla e com finalidades diversas na vigilância da tuberculose no Brasil. Ao mesmo tempo, persistem desafios relacionados à qualidade dos sistemas de informação, à heterogeneidade metodológica dos estudos e à concentração da produção em determinados contextos geográficos e analíticos.

Esta revisão apresenta algumas limitações. Houve dificuldade em coletar dados sobre a quantidade de observações utilizadas nos processos de vinculação e os registros efetivamente pareados, devido à falta de objetividade de alguns artigos ao reportarem essas informações. Muitos artigos apresentaram redação confusa e cálculos amostrais inconsistentes, interferindo diretamente na coleta de informações. Ainda, alguns processos de vinculação de dados tiveram seu percentual de recuperação reduzido após revisões manuais ou aplicação de critérios epidemiológicos relacionados à qualidade dos dados, como inconsistência de datas ou duplicidade de registros.

Além disso, não foi realizada avaliação formal da qualidade metodológica ou do risco de viés dos estudos incluídos, pois o objetivo da revisão foi descrever características metodológicas dos estudos, e não sintetizar estimativas de efeito ou associações epidemiológicas; adicionalmente, havia heterogeneidade dos delineamentos, objetivos analíticos e formas de relato dos procedimentos de vinculação nos estudos incluídos.

## MATERIAL SUPLEMENTAR

Tabela Suplementar 1
